# A Risk-Tiered Validation Framework for Artificial Intelligence in Drug Discovery: From Reproducibility to Clinical Translation

**DOI:** 10.3390/ijms27104349

**Published:** 2026-05-13

**Authors:** Sarfaraz K. Niazi

**Affiliations:** College of Pharmaceutical Sciences, Washington State University, Spokane, WA 99202, USA; niazi@wsu.edu

**Keywords:** artificial intelligence, machine learning, molecular sciences, computational chemistry, protein–ligand prediction, molecular dynamics, drug discovery, validation, regulatory science, ensemble prediction, uncertainty quantification, model-informed drug development, lifecycle governance

## Abstract

Artificial intelligence has advanced from merely predicting static protein structures to modeling equilibrium conformational ensembles. It now concurrently forecasts structure and binding affinity and actively participates in candidate selection during the initial stages of drug discovery. Foundation models introduced between 2024 and 2026, including BioEmu, AlphaFlow, DiG, Boltz-2, Chai-1, NeuralPLexer, and explicit-solvent prediction systems such as SuperWater, have begun to address issues previously identified as fundamental concerns in earlier critiques of AI in drug discovery. Nevertheless, many of these models are presently accessible only as preprints and require validation through independent peer review. Evidence indicates a shift in the primary bottleneck from representation challenges to validation difficulties. However, this transition remains incomplete and heavily dependent on context. The risks associated with AI-enabled drug discovery are increasingly not solely about the models’ capacity to accurately represent ensembles, but also about whether the evidentiary standards used to validate AI-derived predictions keep pace with the rapidity with which these predictions are generated and employed. This article introduces a four-tier validation framework designed to align the extent of computational and experimental evidence with the translational and regulatory risks associated with various artificial intelligence (AI) applications within the molecular sciences. These applications include machine learning (ML) models that analyze sequences, structures, conformational ensembles, protein–ligand complexes, and molecular dynamics trajectories. Tier 1 addresses the internal reproducibility of ML inference when applied to molecular inputs; Tier 2 pertains to the robustness of molecular-science benchmarks such as CASP, CASF-2016, PoseBusters, and OpenFE; Tier 3 involves prospective experimental validation against biophysical and biochemical measurements; and Tier 4 encompasses clinical and translational calibration within physiologically based pharmacokinetic (PBPK) and quantitative systems pharmacology (QSP) frameworks. This validation hierarchy functions as an explicit conceptual guide, serving as a framework rather than a regulatory requirement. It is firmly grounded in established principles derived from ICH Q8/Q9/Q10, the FDA model-informed drug development (MIDD) approach, the EMA reflection paper on AI in the medicinal product lifecycle, and the EU AI Act. The manuscript further incorporates recent evidence from ensemble-aware AI, prospective docking, free-energy campaigns, and clinical-stage AI-derived candidates. It concludes with specific recommendations pertaining to lifecycle governance, uncertainty reporting, and the adoption of harmonized evidentiary templates for AI/ML applications in the molecular sciences.

## 1. Introduction

Public discourse on artificial intelligence in drug discovery has progressed through three distinct phases over a period of less than 10 years. The initial phase, approximately from 2018 to 2021, was predominantly characterized by advancements in structure prediction, exemplified by AlphaFold2 and RoseTTAFold, which demonstrated that deep learning techniques could accurately determine protein tertiary structures, approaching the geometric precision of experimental methods for numerous well-folded globular proteins [1,2]. During this period, the AlphaFold Protein Structure Database expanded its predictive coverage to include hundreds of millions of sequences [3]. The subsequent phase, roughly from 2022 to 2024, saw an expansion of scope, with diffusion-based generative models facilitating de novo protein design [4,5], and protein language models revealing that structural information inherently exists within large-scale sequence spaces [6,7]. Additionally, AlphaFold3 extended its predictive capabilities to multi-component complexes, including those with small-molecule ligands [8]. Presently, the third phase is characterized by ensemble emulation, integrated structure–affinity prediction, and the explicit incorporation of experimental data. Foundation models developed between 2024 and 2026 are reported to generate approximate equilibrium ensembles whose relative free energies between resolved metastable states and predicted effects of point mutations on folding stability align with millisecond-scale molecular dynamics and experimental reference data to within approximately 1 kcal/mol on in-domain test systems under controlled benchmarking conditions. The original authors caution that transferability beyond the training distribution remains uncertain [9,10]. These models have also been demonstrated to approach the accuracy of free-energy perturbation (FEP) methods on select benchmark datasets while being significantly less computationally intensive [11,12], and to localize crystallographic water molecules within sub-ångström accuracy on structures like their training data [13].

These advancements have fundamentally transformed the practical considerations confronted by regulators, developers, and clinical pharmacologists. AI systems have demonstrated their capacity to generate pharmacologically relevant hypotheses at scale in preclinical environments; however, the evidentiary standards required to use these predictions for decisions with therapeutic and regulatory consequences remain unresolved. This issue is further accentuated by three principal observations. Firstly, the proliferation of AI models in the discovery phase outpaces the development of evaluation benchmarks; retrospective enrichment is an unreliable predictor of prospective performance, and failures in prospective applications are markedly underreported [14]. Secondly, AI systems exhibit distinct modes of failure, such as mode collapse, distributional shift, and miscalibrated uncertainty, that are insufficiently addressed by validation procedures originally designed for analytical methods or deterministic simulation software [15,16]. Thirdly, the regulatory landscape is evolving swiftly, exemplified by recent draft guidance from the U.S. Food and Drug Administration (FDA) [17], a reflection paper issued by the European Medicines Agency (EMA) [18], and the EU AI Act [19], all emerging within a single regulatory cycle.

This article delineates a risk-tiered validation framework that aligns the burden of evidence with the translational consequences across the entire spectrum of artificial intelligence and machine-learning applications in drug discovery. It expressly focuses on the molecular-science layer at which contemporary AI/ML systems operate, namely, sequence-based representation learning, static and ensemble structure prediction, protein–ligand complex and affinity prediction, generative molecular and protein design, machine-learned interatomic potentials, and ML-augmented analysis of molecular dynamics trajectories. The framework is not intended as a regulatory standard; rather, it serves as a conceptual scaffold derived from established practices in computational chemistry benchmarking, pharmaceutical development validation, and risk-based regulatory governance. Its primary aim is to facilitate communication among developers, reviewers, and regulators regarding AI evidence by employing a shared vocabulary that clearly distinguishes between exploratory use, where lenient validation is appropriate, and decision-critical use, where rigorous validation is warranted. By anchoring each tier to the class of molecular-science output a model produces, whether a single structure, an ensemble, a binding pose, a ΔG estimate, or a trajectory-derived kinetic parameter, the framework is designed to be directly actionable across AI/ML methods employed in computational chemistry, structural biology, and early drug-discovery pipelines.

Methodological Transparency: This article constitutes a narrative critical review. Literature was identified through targeted searches of PubMed, Web of Science, and preprint archives (arXiv and bioRxiv) covering the period from 2018 through early 2026. These findings were supplemented by citation chaining from recent review articles and by the author’s tracking of recent preprint releases in the fields of structural biology and computational drug discovery. Recent foundation models were included only when primary publications or peer-reviewed journal versions were available; preprint-only sources are designated as such in the reference list and identified in the text. Evidence is interpreted hierarchically throughout: peer-reviewed prospective studies are prioritized over retrospective benchmark analyses, which are in turn prioritized over preprint reports. Quantitative performance claims derived from preprints are reported in the language used by the original authors and should be re-evaluated upon the publication of peer-reviewed versions. The synthesis presented reflects the author’s interpretation of the existing literature, does not claim to be a systematic review, and does not represent the stance of any regulatory authority.

The manuscript is organized as follows:

- Section 2 summarizes the contemporary AI landscape relevant to drug discovery, emphasizing the ensemble-aware, affinity-aware, and solvent-aware models that now define the state of the art.
- Section 3 identifies the gaps that persist after these advances: prospective reliability, kinetic predictability, chemical validity, and propagation of molecular-scale uncertainty to clinical outcomes.
- Section 4 introduces the four-tier validation ladder and maps specific use cases to each tier.
- Section 5 develops the regulatory alignment of the framework, drawing on FDA MIDD, ICH Q8/Q9/Q10, EMA guidance, and the EU AI Act.
- Section 6 illustrates the framework with clinical-stage case studies, including both successes and failures.
- Section 7 offers concluding recommendations for lifecycle governance and harmonization.

## 2. The Contemporary AI Landscape in Drug Discovery

### 2.1. Why Ensembles Matter

**Figure 1:**
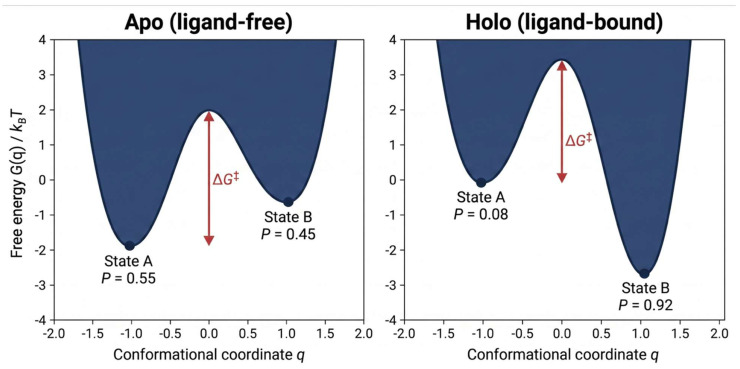
The conformational free-energy landscape before and after ligand binding. In the apo state (**left**), two minima are separated by an activation barrier ΔG^‡^; Boltzmann populations are determined by the depth of each basin. Ligand binding (**right**) decreases the free energy of one basin and shifts the population towards near-complete occupancy of the bound state. Static AI structure prediction typically identifies only the dominant minimum; ensemble-aware methodologies approximate the entire distribution.

Biological function originates from conformational ensembles governed by Boltzmann statistics, rather than from individual static structures [20,21]. At equilibrium, the probability of state Sᵢ is expressed as P(Sᵢ) = exp(−Gᵢ/kBT)/Σⱼ exp(−Gⱼ/kBT), and observable properties are ensemble averages ⟨A⟩ = Σ A(Sᵢ) P(Sᵢ). Ligand binding modifies this distribution according to ΔG_bind = −RT ln K_d [22,23]. Kinases undergo DFG-motif rearrangements that determine the binding modes of type-I and type-II inhibitors [24]; G-protein-coupled receptors (GPCRs) exhibit transmembrane helix rearrangements that stabilize distinct signaling states [25,26]; intrinsically disordered proteins demonstrate considerable conformational heterogeneity [27,28]. Figure 1 schematically illustrates how ligand binding redistributes populations across a multi-minimum free-energy landscape.

Over the past decade, artificial intelligence models have faced criticism for generating only the minimal necessary output, while neglecting the ensemble perspective [29,30]. This critique prompted a significant period of innovative modeling efforts from 2023 to 2026. Subsequently, the emergence of ensemble-aware, affinity-aware, and solvent-aware models now represents the forefront of the field. The following section provides an overview of these advancements.

### 2.2. Structure and Complex Prediction

AlphaFold2, RoseTTAFold, and ESMFold have established deep learning as the dominant paradigm for monomeric protein structure prediction [1,2,6]. AlphaFold3 has expanded this capability to include multi-component complexes comprising small-molecule ligands, nucleic acids, and post-translational modifications [8]. Recent open foundation models such as Boltz-1, Boltz-2, and Chai-1 have not only reproduced but, in several respects, exceeded the capabilities of AlphaFold3, while making their weights, training code, and inference pipelines available under permissive licenses [11,12,31]. Specifically, on the 2024–2025 PDB holdout used for benchmarking complex prediction, Boltz-2 demonstrates notable improvement over AlphaFold3, particularly in challenging categories such as antibody–antigen complexes, and provides inference-time steering potentials (Boltz-2x) that help enforce physically plausible bond geometries [11].

### 2.3. Ensemble Emulation: Generative Models Trained on Molecular Dynamics

The most notable recent advancement regarding the thesis of this manuscript is the emergence of deep learning systems that generate approximate equilibrium ensembles rather than single-point predictions. BioEmu, introduced by Microsoft Research in 2024 and published in Science in 2025 [9], exemplifies a diffusion-based generative model trained on over 200 milliseconds of molecular dynamics (MD) trajectories combined with experimental stability measurements. It proficiently samples statistically independent structures from the approximate equilibrium distribution of a protein monomer at a rate of thousands of samples per GPU-hour, capturing cryptic binding-pocket formation, local unfolding, and domain rearrangements. The reported accuracy of approximately 1 kcal/mol attributed to BioEmu is provided by the original authors specifically for two scoped settings on in-domain test systems under the discussed benchmarking conditions: agreement with relative free energies between resolved metastable conformational states sampled from millisecond-scale molecular dynamics, and prediction of the effects of single-point mutations on protein folding stability against experimental reference data. This figure does not encompass ligand binding affinity, absolute folding free energy, or kinetic rate constants, and the original authors explicitly caution that transferability beyond the training distribution remains uncertain, particularly for proteins, sequence regions, and environmental conditions dissimilar to those represented in the training corpus. AlphaFlow integrates the AlphaFold2 architecture with flow matching to generate structural ensembles [10], and the Distributional Graphormer (DiG) extends related concepts to broader molecular systems [32].

These models are not intended to replace Molecular Dynamics (MD) simulations or advanced sampling techniques; rather, their primary purpose is to reduce the computational cost of generating representative samples from the Boltzmann distribution. As a result, inquiries that previously necessitated weeks of specialized simulation, such as estimating the approximate population of a cryptic pocket or ranking the thermal stability of mutants, can now be completed within minutes. Nonetheless, it is crucial to acknowledge that the transferability of these models beyond their training distribution remains uncertain. For example, BioEmu does not intrinsically account for ligand interactions, membrane environments, or temperature-dependent behaviors [9,33]. Therefore, ensemble emulators should be considered as rapid, approximate samplers whose outputs demand the same rigorous validation as any other AI-based predictions.

### 2.4. Joint Structure–Affinity Foundation Models

Boltz-2 is distinguished not only as a model capable of ensemble applications but also as the inaugural open foundation model in structural biology designed to predict binding affinity end-to-end with competitive accuracy [11]. In the CASP16 affinity track, a blind benchmarking exercise involving 140 protein–ligand pairs across two targets, Boltz-2’s out-of-the-box deployment was preliminarily documented in a preprint and was observed to outperform submitted entries, including methods that entailed extensive custom preparation over several weeks; comprehensive peer-reviewed analyses of the CASP16 affinity track are forthcoming. On the OpenFE benchmark dataset, preliminary reports indicate that Boltz-2 approaches the performance of OpenFE’s relative free-energy perturbation (RFEP) method while being approximately 3 orders of magnitude faster under the specified benchmark conditions [11]. The integration of Boltz-2 with a generative molecular model has demonstrated efficacy as a pipeline for discovering diverse, synthesizable, high-affinity binders, with results validated through absolute free-energy perturbation (ABFE) calculations [11]. Chai-1, developed by Chai Discovery, in conjunction with NeuralPLexer, occupies comparable positions within this domain [31,34].

These models exemplify advances within their respective categories compared with earlier AI docking systems. Past methodologies such as DiffDock, EquiBind, and TANKBind [35,36,37] treated the receptor as largely static and were criticized for producing poses that lacked physical plausibility [38]. PoseBusters analyses revealed significant rates of chemical validity failure across several techniques (including bond lengths, clashes, ring planarity) on challenging test sets, despite achieving competitive RMSD values [38]. The current generation of models, namely Boltz-2x, Chai-1, and NeuralPLexer, integrates joint receptor flexibility, physicality potentials, and inference-time constraints, leading to considerably higher chemical validity pass rates on matched test sets [11,31]. It is important to emphasize that some of the most compelling empirical claims regarding Boltz-2 are based on preprints that have not yet undergone peer review; therefore, the conclusions presented herein should be reevaluated if the peer-reviewed versions yield substantively different findings.

### 2.5. Explicit-Solvent AI and Water-Aware Binding

A longstanding critique of artificial intelligence (AI) scoring functions has been their reliance on implicit-solvent assumptions, which consequently underrepresent the enthalpic and entropic effects associated with water displacement from binding sites [39]. This issue has been partially addressed through the development of dedicated water-prediction models. SuperWater, a scoring-based diffusion model trained on crystal structures with resolved water molecules, localizes bound waters within 0.3 ± 0.06 Å of their experimental positions for crystallographically identified waters. It surpasses earlier tools such as GalaxyWater-CNN and HydraProt in terms of the precision–coverage trade-offs on reported benchmarks; however, its applicability to dynamic hydration processes and binding thermodynamics remains unvalidated [13]. Grid-based methods, including WaterMap and inhomogeneous solvation theory, continue to be extensively utilized for strategic analysis of water displacement [40]. The practical implication is that solvent-aware prediction, once regarded as a frontier limitation, has become increasingly accessible as a modular component bridging structure prediction and free-energy scoring. Despite this progress, it is not yet a standard element in industrial pipelines, and integrating such methods with thermodynamic workflows remains an active area of development.

### 2.6. Machine-Learned Potentials as a Distinct Methodological Category

Machine-learned force fields (MLFFs) constitute a distinct methodological category from the generative ensemble models delineated earlier. MLFFs, including MACE, Allegro, ANI, and SchNet, approximate quantum-mechanical potential energy surfaces using neural networks trained on electronic structure calculations [41,42,43]. Their application within drug discovery pipelines predominantly involves substituting classical force fields in regions where phenomena such as polarization, charge transfer, or reactive chemistry are mechanistically significant. Quantum mechanics/molecular mechanics (QM/MM) methodologies are utilized to generate high-fidelity training labels for MLFFs and remain the benchmark for hybrid inference, especially in reactive or metal-coordinated active sites [44,45]. MLFFs are orthogonal to and can be integrated with enhanced sampling and ensemble emulation techniques: for example, a MACE potential may be employed within molecular dynamics (MD) or metadynamics trajectories to inform a BioEmu-style generative model, or alternatively, may be directly used in inference-time refinement of a predicted complex.

### 2.7. Sampling, Analysis, and Their Distinct Roles

Four concepts that are occasionally conflated warrant clear delineation. Enhanced sampling techniques, including replica-exchange molecular dynamics (MD), metadynamics, well-tempered metadynamics, and on-the-fly probability enhanced sampling (OPES), modify the sampled distribution to overcome kinetic barriers [46,47]. Methods for collective-variable (CV) discovery, such as TICA, VAMPnets, SPIB, and deep-LDA, identify low-dimensional order parameters and can be used both to analyze MD trajectories and to define bias coordinates for enhanced sampling [48,49]. Markov state models (MSMs) constitute a kinetic analysis framework that discretizes conformational space and estimates transition probabilities from trajectories generated by any sampling method [50,51]. Machine Learning Force Fields (MLFFs), as previously discussed, replace the underlying potential energy function. These four methodologies are mutually complementary; none is a subset of any of the others. Precise differentiation among them is essential, as review articles that group them under a single heading may misrepresent the modular architecture of modern computational workflows.

### 2.8. Training Datasets That Go Beyond the PDB

Benchmark training data has also advanced considerably. The MISATO dataset combines semi-empirical QM-refined structures with explicit-solvent molecular dynamics trajectories, amounting to approximately 170 microseconds across roughly 20,000 protein–ligand complexes sourced from PDBbind [52]. MISATO provides dynamics-aware training labels, such as atomic adaptability and binding-site flexibility, and has been utilized to train relational graph neural networks that integrate both static and dynamic signals for affinity prediction and binding-site identification [52]. The ATLAS database systematically compiles MD-derived flexibility data for thousands of protein chains [53]. PLINDER addresses dataset leakage by implementing more rigorous protein–ligand clustering procedures and provides evaluation splits that help reduce inflated benchmark metrics [54]. These resources partially alleviate but do not fully eliminate concerns that artificial intelligence training data inherit experimental biases toward dominant, cryogenically frozen, dilute-buffer conformations [55,56]; residual biases still require careful assessment for each downstream application.

Table 1 summarizes the categories of contemporary AI systems discussed in this section and the representative models in each category.

## 3. Gaps That Persist After Ensemble-Aware AI

### 3.1. The Prospective–Retrospective Gap

**Figure 2:**
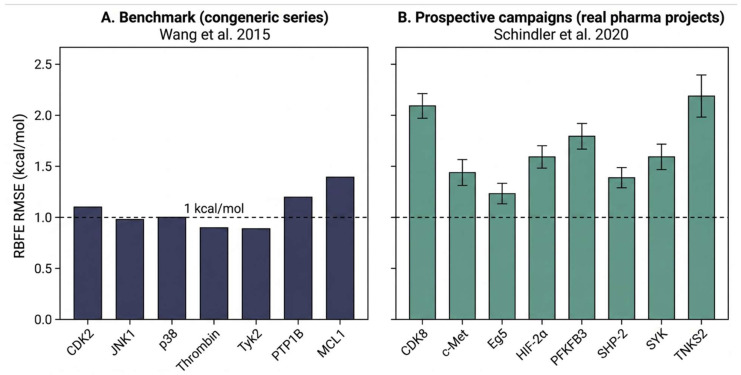
RBFE prediction accuracy across benchmark and prospective settings. Panel **A** presents RMSE values from the Wang et al. [57] congeneric-series benchmark across seven well-studied targets; most values are near or below 1 kcal/mol. Panel **B** illustrates RMSE values from Schindler et al.’s [58] survey of prospective RBFE campaigns in active pharmaceutical projects; these values are higher (1.2–2.2 kcal/mol) and exhibit greater variability. The two studies are retained as illustrative reference points because they remain the most widely cited curated and prospective comparators in the field; absolute accuracy on congeneric benchmarks has improved in subsequent work, including harmonized benchmarking practices recommended by Hahn et al. [59] and recent foundation-model evaluations [11,60], yet the directional gap between curated benchmark and prospective campaign performance persists. The dashed line indicates 1 kcal/mol as a conventional reference. The observed discrepancy reflects a distributional shift between curated training datasets and the real-world chemistry of projects. Data are re-plotted from Wang et al. [57] and Schindler et al. [58].

Retrospective benchmark performance is an unreliable predictor of prospective campaign performance. This is not a novel observation [14], but the gap has become quantitatively sharper as more prospective data have accumulated. Figure 2 illustrates the phenomenon for relative binding free-energy (RBFE) calculations. On the Wang et al. [57] benchmark of congeneric ligand series across well-studied targets, RMSE values cluster near or below 1 kcal/mol [57]. On Schindler et al.’s [58] analysis of active pharmaceutical RBFE campaigns, RMSE values across eight prospective projects range from 1.2 to 2.2 kcal/mol with considerably greater variability [58]. More recent benchmark and prospective studies, including the harmonized practices recommended by Hahn et al. [59] and successive Boltz-2 and OpenFE evaluations [11,60], document that absolute RBFE accuracy on curated congeneric series has continued to improve, with several modern protocols reporting RMSE values closer to 1 kcal/mol on broader test sets than were available a decade ago. The directional gap, however, persists: prospective campaigns on novel chemotypes, scaffold hops, and previously uncharacterized targets continue to display higher and more variable error than the curated benchmarks used during method development. The gap is driven by distributional shift: novel chemotypes, non-congeneric scaffold hops, charged species, and targets with limited parameterization all degrade performance in ways that benchmarks cannot fully anticipate (Figure 2).

The consistent pattern observed across AI docking indicates that the anticipated performance of AI docking methodologies on time-split test sets and novel sequences is markedly inferior to their performance on the curated retrospective benchmarks initially used to publish these methods [38,61]. Independent systematic assessments of AI-generated molecular libraries have documented that a considerable proportion of de novo designs fail to satisfy basic synthetic accessibility or pharmacokinetic criteria, despite demonstrating high predicted potency [62,63]. These findings do not imply that ensemble-aware AI is not a significant advancement; rather, they highlight the importance of documenting the circumstances under which such progress is applicable and of generating prospective evidence prior to employing predictions in decision-making processes with translational implications.

### 3.2. Chemical and Physical Validity as a Separate Axis

The PoseBusters framework underscores a dimension orthogonal to affinity or pose accuracy: chemical validity [38]. A docked pose exhibiting a favorable predicted score and low RMSD relative to the crystal reference may nevertheless contravene bond-length constraints, present clashes, or assume improbable ring conformations. Such instances represent a validity failure rather than an accuracy failure, thereby exerting a distinct impact on downstream workflows: a chemically invalid pose cannot be effectively optimized by subsequent medicinal chemistry efforts, irrespective of its scoring function. Current state-of-the-art models incorporate physicality steering mechanisms (e.g., Boltz-2x’s enforcement of bond-geometry priors); however, these measures do not eradicate the issue [11]. Consequently, validity must be reported separately from accuracy and uncertainty in any rigorous evaluation.

### 3.3. Binding Kinetics and Residence Time

Equilibrium affinity is necessary but not sufficient for clinical efficacy. Drug–target residence time (/koff) correlates with in vivo efficacy across several therapeutic classes, particularly when target turnover is slow relative to drug clearance [64,65]. Dissociation rates are influenced by transition-state barriers and conformational gating events that are not captured by static structural prediction and are only partially represented by ensemble emulation. Markov state modeling facilitates the estimation of kinetic rate constants from molecular dynamics trajectories [50,51]; however, convergence necessitates sufficient sampling. Furthermore, experimental validation using surface plasmon resonance or stopped-flow kinetics remains essential. While artificial intelligence-based kinetic prediction is an active area of research, it has not yet reached the level of prospective reliability currently achieved for equilibrium structures or affinities.

### 3.4. Uncertainty Quantification and Distributional Shift

Calibrated uncertainty is imperative whenever predictive models underpin decision-making processes. Methods such as deep ensembles and Bayesian approximations improve uncertainty estimation compared to individual deterministic network models [15,66]. The expected calibration error (ECE) and the Brier score are metrics for assessing the reliability of probabilistic predictions [67,68]. However, under conditions of distributional shift, calibration quality can significantly deteriorate: a model that exhibits well-calibrated confidence intervals within the training distribution may produce overconfident predictions when encountering out-of-distribution chemotypes or targets [16,69]. This issue is particularly relevant in generative and de novo design applications, where the explicit aim is to generate molecules or proteins that extend beyond previously characterized chemical or sequence spaces. It is essential that the reporting of uncertainty differentiates between epistemic uncertainty (stemming from limited training data and reducible through additional data) and aleatoric uncertainty (originating from inherent thermodynamic variability and irreducible), ensuring that these uncertainties are evaluated separately in both in-distribution and out-of-distribution contexts.

### 3.5. Propagation from Molecular to Clinical Scales

A prediction of ΔG_bind, accompanied by the stated uncertainty, constitutes the initial stage rather than the culmination of translational analysis. Uncertainty at the molecular level propagates through physiologically based pharmacokinetic (PBPK) models, pharmacodynamic models, population pharmacokinetic (PK) models, and trial-simulation frameworks. Physiologically based pharmacokinetic models depict tissues as compartments characterized by blood flow, volume, partitioning, and clearance parameters [70]. Quantitative systems pharmacology (QSP) links molecular parameters to pathway-level and clinical outcomes [71]. When AI-derived parameters are incorporated into these frameworks, their uncertainty must be propagated rather than substituted by point estimates. Bayesian updating enables the revision of computational priors as clinical data accumulate during early-phase trials [72]. Bayesian adaptive trial designs explicitly incorporate such updating into dose selection and interim decision-making processes [73]. The translational pipeline functions as a sequential chain; consequently, the weakest link in the uncertainty characterization determines the confidence level in the final clinical prediction. Hybrid machine learning–mechanistic approaches, in which AI-derived parameter distributions are propagated through PBPK and QSP models employing Monte Carlo sampling, Bayesian hierarchical modeling, or computationally efficient surrogates, provide a pragmatic route for such propagation. Recent regulatory activities explicitly align with these probabilistic methodologies, including the FDA draft guidance on Bayesian methodology in clinical trials released in January 2026 [74] and the EMA’s 2025 literature review on uncertainty quantification for regulatory modeling [75].

## 4. A Risk-Tiered Validation Ladder for AI in Drug Discovery

AI systems in drug discovery operate across a broad spectrum of translational contexts. A model employed to generate hypotheses during exploratory screening has different implications than one whose output directly informs clinical dose determination. Applying a uniform validation standard to all scenarios risks overburdening exploratory research or inadequately validating applications critical to decision-making. This section introduces a four-tier validation framework that correlates the depth of evidence with the level of translational risk. It is a conceptual model, not a regulatory criterion. It is not endorsed by any regulatory agency and should not be presented to regulators as established guidance. Its objective is to facilitate structured discussions regarding evidentiary requirements.

**Figure 3:**
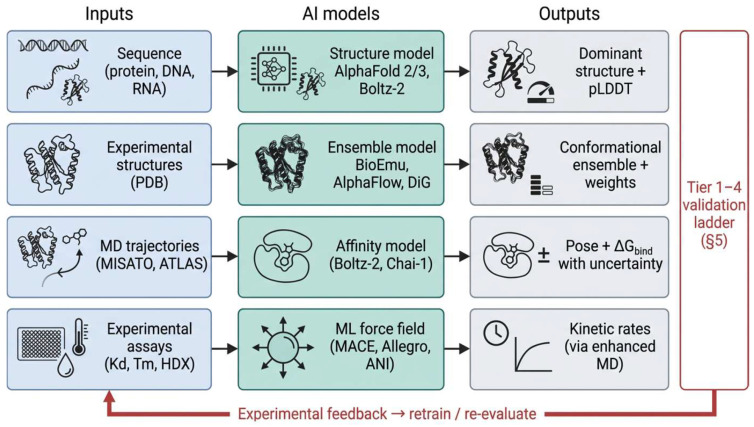
Ensemble-aware AI-driven workflow for drug discovery. Inputs, including sequences, experimental structures, molecular dynamics trajectories, and experimental assays, are processed through modern artificial intelligence models (such as structure prediction, ensemble emulation, joint affinity prediction, and machine-learned force fields). These models generate outputs ranging from predominant structures to conformational ensembles with associated weights, ΔG_bind_ estimates with quantified uncertainties, and kinetic rate constants. All outputs are subjected to a risk-tiered validation protocol outlined in Section 4, with experimental feedback mechanisms completing the loop for model retraining and re-evaluation.

**Figure 4:**
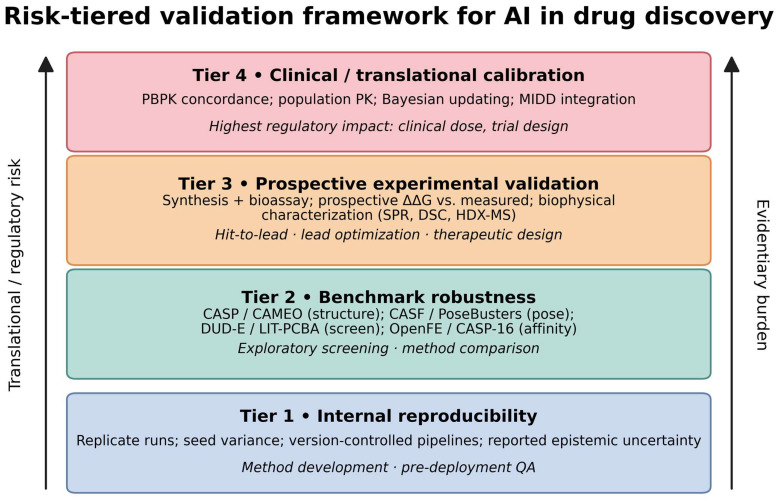
The four-tier validation ladder. Tier 1 (internal reproducibility) establishes that predictions are stable computational outputs rather than stochastic artifacts. Tier 2 (benchmark robustness) evaluates performance against community benchmarks under controlled conditions. Tier 3 (prospective experimental validation) tests predictions against independent experimental data not used in training. Tier 4 (clinical and translational calibration) integrates predictions into PBPK/QSP frameworks and assesses concordance with population-scale clinical observations. Each ascending tier introduces additional sources of uncertainty and requires proportionally more evidence; no tier is sufficient in isolation.

The framework integrates practices from computational chemistry benchmarking [76,77], pharmaceutical development validation [78], and risk-based regulatory governance [17,18,79]. Figure 3 places it in the context of the modern AI workflow, and Figure 4 illustrates the ladder directly.

### 4.1. Tier 1: Internal Reproducibility

Prior to engaging in external benchmarking or experimental validation, it is essential that AI models demonstrate computational reproducibility. Contemporary architectures incorporate stochastic components, such as random weight initialization, dropout, and sampling-based inference, that can yield different outputs across multiple executions. Tier 1 validation necessitates comprehensive documentation of replicate inference runs, including the reported prediction variance, convergence diagnostics during training, and artifacts, including datasets, preprocessing techniques, hyperparameters, and software versions, adequate to facilitate result reproduction across various computational environments. At this stage, the emphasis is on computational stability rather than biological validity. Failures at this level typically indicate inadequate training convergence, unstable architectural configurations, or pipeline fragility.

Minimum evidence for Tier 1 encompasses the replication of inference runs with reported seed variance, the execution of convergence diagnostics for training procedures, the maintenance of a version-controlled repository, the utilization of containerized or portable runtime environments, the documentation of policies pertaining to random seeds, and the reporting of epistemic uncertainty on held-out inputs.

### 4.2. Tier 2: Benchmark Robustness

Once computational stability has been established, models should be evaluated against community benchmarks designed to measure performance in specified domains. In the fields of structural biology and computational chemistry, several widely accepted benchmarks serve this purpose. CASP and CAMEO assess structure prediction using GDT-TS and lDDT [76,80]. CASF-2016 evaluates scoring functions against experimental binding affinities, with leading methods attaining Pearson correlations of 0.80–0.86 [77,81]. DUD-E and the more recent LIT-PCBA evaluate enrichment in virtual screening [82,83]. PoseBusters assesses the chemical validity of predicted poses [38]. The CASP16 affinity track offers a blind evaluation of affinity prediction for protein–ligand pairs [84]. OpenFE benchmarks relative free-energy calculations [60].

Strategies for dataset partitioning should aim to reduce information leakage. Preferring scaffold-based or cluster-based splitting over random splitting is advisable; moreover, a time-split evaluation, training on data prior to 2022, and testing on complexes formed after 2022, serves as a more rigorous criterion that reveals models to authentic distribution shifts [38]. Absent such precautions, performance estimates may be artificially inflated, as demonstrated by analyses of dataset bias in AI-driven drug discovery [14].

The minimum evidence required for Tier 2 encompasses performance on at least one community benchmark aligned with the task; assessment through scaffold- or time-split methods with comprehensive reporting of leakage controls; chemical validity metrics for models generating atomic coordinates; calibration metrics such as Expected Calibration Error (ECE) and reliability diagrams for models providing probabilistic outputs; and thorough documentation of training data provenance and identified biases.

Achieving success at Tier 2 is a necessary condition; however, it is not sufficient on its own. Benchmark performance varies across contexts, and extending conclusions beyond the benchmark domain, such as to novel target classes, chemotypes, or biological contexts outside the training distribution, cannot be confidently supported solely by Tier 2 evidence.

### 4.3. Tier 3: Prospective Experimental Validation

Prospective validation involves assessing predictions against experimental data not used during model development and introduces sources of uncertainty that curated benchmarks cannot capture, such as experimental noise, biological variability, and previously unencountered chemical scaffolds. In the context of generative molecular design, prospective validation entails synthesizing the predicted compounds and measuring their biological activity in vitro. For affinity-prediction models, this involves comparing the predicted and measured ΔG values for compounds designed and synthesized after the model is trained. Regarding protein-design models, it includes expressing the designed proteins, assessing thermal stability, characterizing aggregation behaviors, and validating target binding through orthogonal methods such as surface plasmon resonance (SPR), biolayer interferometry (BLI), differential scanning calorimetry (DSC), and hydrogen–deuterium exchange mass spectrometry (HDX-MS) [85,86].

Prospective validation refers to the stage at which computational forecasts are first tested against actual biological complexity. Discrepancies observed between retrospective benchmark outcomes and prospective experimental data often underscore biases within the dataset, inadequate representation of receptor flexibility, or fundamental limitations of force-field parameterization [14,58]. Publication bias is a well-documented concern at this juncture: unsuccessful prospective AI efforts are markedly underreported compared to successful instances, thereby fostering an exaggerated perception of prospective reliability [14,87,88].

The minimum requirements for Tier 3 include prospective predictions recorded prior to the experimental readout; orthogonal experimental validation, such as at least two independent biophysical or functional assays where feasible; reported hit rate or prediction accuracy compared against prospective experimental measurements; documentation of both failed and successful predictions; and the preservation of negative results in internal archives, even if they are not published.

### 4.4. Tier 4: Clinical and Translational Calibration

The highest tier is designated when predictions generated by artificial intelligence influence clinical decision-making or regulatory strategies. At this level, model outputs must demonstrate concordance with translational pharmacology and clinical observations. Validation efforts extend beyond assessing molecular prediction accuracy to encompass integration with physiologically based pharmacokinetic (PBPK) models, pharmacokinetics/pharmacodynamics (PK/PD), quantitative systems pharmacology (QSP), and population pharmacokinetic (population-PK) modeling frameworks [70,71,89]. When artificial intelligence models produce parameters that serve as inputs to these systems, such as binding affinities, estimates of metabolic stability, and receptor-activation probabilities, the associated uncertainty must be propagated through subsequent models. Consequently, validation requires that predictions derived from artificial intelligence produce exposure and response profiles aligned with clinical observations, and that the predictive uncertainty observed at the clinical level can be traced back to the molecular predictions that generated it.

Population pharmacokinetic modeling incorporates interindividual variability through mixed-effects models: CLᵢ = CLtyp · exp(ηᵢ), where ηᵢ represents between-subject random effects. Bayesian updating is applied according to p(θ|D) ∝ p(D|θ) · p(θ), thereby enabling the revision of computational priors as clinical data are accumulated [72]. In adaptive Bayesian trial designs, the posterior probability of a clinically meaningful treatment effect, P(δ > τ|Dₖ), is updated at each interim analysis, thus permitting dose modifications or early termination based on real-time evidence [73]. Priors derived from artificial intelligence must remain subordinate to empirical data and should be transparently documented as informative starting points rather than immutable constraints.

The minimum evidence required for Tier 4 encompasses documented contextual usage, the integration of Physiologically Based Pharmacokinetic (PBPK) or Quantitative Systems Pharmacology (QSP) models with uncertainty propagation, concordance of population pharmacokinetics (PK) when human data are accessible, Bayesian updating with explicitly defined prior specifications, a comprehensive lifecycle monitoring plan that incorporates drift detection thresholds and pre-specified retraining triggers, and documented regulatory interactions in compliance with relevant jurisdictions.

### 4.5. Mapping Use Cases to Tiers

Table 2 presents representative artificial intelligence use cases along with their respective minimum validation tiers. These assignments are heuristic and context-dependent; practitioners are advised to consult primary literature and pertinent regulatory guidance relevant to their specific circumstances. The table does not serve as a regulatory standard and should not be submitted to agencies as a compliance artifact.

### 4.6. Interpretation and Limits of the Ladder

The validation ladder exemplifies a continuum of evidentiary integration rather than a rigid checklist. Achieving benchmark success without prospective validation may indicate dataset bias; attaining prospective success without computational reproducibility could suggest unstable pipelines; and clinical concordance without traceable molecular validation might signify coincidence rather than underlying mechanisms. Reliable AI-enabled drug discovery requires integrating evidence across computational, experimental, and translational domains.

Ensemble-aware methodologies entail significantly higher computational costs than static screening techniques. Executing a BioEmu ensemble simulation or a Boltz-2 affinity evaluation requires GPU resources that, while minimal relative to a CASP-era molecular dynamics campaign, remain considerable compared to traditional docking procedures. For targets characterized by conformational rigidity, well-crystallized structures, and supported by comprehensive structure–activity relationship (SAR) data, static docking utilizing machine-learned scoring functions may achieve predictive accuracy comparable to ensemble approaches, while incurring markedly lower computational expenses. Consequently, this classification should not be construed as mandating the exclusive application of ensemble techniques in such scenarios; rather, the selection of methodologies should be tailored according to the target’s conformational flexibility, the robustness of available structural data, and the pertinent translational risk category.

Ensemble methods also present specific failure modes. Molecular dynamics (MD)-based sampling is susceptible to systematic errors in force fields that consistently generate biased free-energy estimates, regardless of the sampling duration. Documented cases include errors in partial-charge assignment for non-standard ligands and in metal coordination geometries [44,90,91]. Metadynamics may overfit the selection of collective variables, leading to seemingly converged free-energy profiles that do not accurately represent biologically relevant transitions [47]. Markov state models require sufficient trajectory data to adequately populate pertinent states; for transitions occurring over timescales longer than the available simulation durations, the transition rates derived from MSMs may be unreliable despite apparent convergence [51]. Ensemble emulators such as BioEmu have failed when applied to proteins substantially dissimilar to their training distributions [9]. In all these cases, the marginal advantage of increased ensemble complexity over simpler techniques may be negligible or even detrimental, particularly during the initial stages of the discovery process, where the quality of targets and binding sites remains uncertain. The implementation of ensemble-aware methodologies should be accompanied by predefined convergence criteria and explicit assessments of whether the computational expenditure is justified by the anticipated reduction in translational uncertainty at the respective stage.

## 5. Regulatory Alignment and Lifecycle Governance

### 5.1. The Regulatory Landscape, Mid-2025 to 2026

The FDA’s draft guidance from January 2025 regarding the use of artificial intelligence to support regulatory decision-making for drugs and biological products (non-binding) delineates a risk-based framework that emphasizes the documentation of model architecture, training data provenance, validation procedures, and lifecycle management. The EMA’s 2024 reflection paper on AI utilization in the medicinal product lifecycle, jointly published by the Committee for Medicinal Products for Human Use (CHMP) and the Committee for Medicinal Products for Veterinary Use (CVMP), describes risk-proportionate oversight and transparency requirements applied throughout discovery, development, and post-market surveillance. The EU AI Act (Regulation 2024/1689) [92] establishes harmonized obligations for high-risk AI systems, including those utilized in healthcare applications, with phased enforcement extending through 2026; its scope broadly encompasses high-risk AI systems, with specific implications for drug discovery validation remaining under development. Governance frameworks from Japan’s Pharmaceuticals and Medical Devices Agency (PMDA), China’s National Medical Products Administration (NMPA), Australia’s Therapeutic Goods Administration (TGA), and the World Health Organization (WHO) ethics guidelines offer additional structures of varying detail [93]. The FDA’s authorization of an AI-based drug development tool for MASH clinical trials, announced in 2025, exemplifies the extensive validation infrastructure required for a specific context of use and provides an early, tangible example of the evidence package anticipated by regulatory agencies. The FDA’s draft guidance from January 2026 regarding the application of Bayesian methodology in clinical trials for drugs and biological products advances this trajectory by formalizing expectations concerning principled prior specification [94], prospective evaluation of operating characteristics, and computational transparency. Although primarily aimed at clinical trial inference rather than AI model qualification, this guidance bears direct implications for Tier 4 application of AI-derived priors in model-informed decision-making and complements the EMA’s 2025 review of literature on uncertainty quantification in regulatory modeling.

### 5.2. Risk-Proportionate Evidentiary Reasoning

Risk-proportionate validation is a well-established principle within pharmaceutical science, as articulated in ICH Q8 (pharmaceutical development), Q9 (quality risk management), and Q10 (pharmaceutical quality system), and it is further extended through the FDA model-informed drug development (MIDD) framework [78,79,95]. The principle states that the evidentiary depth should correspond proportionally to the level of uncertainty and the potential consequences associated with the decision being supported. Independent regulatory scholarship has applied similar reasoning to the qualification of computational models: Zhao et al. [95] and Jean et al. [72] analyze physiologically based pharmacokinetic (PBPK) model acceptance criteria in terms that scale with the clinical implications of the model output, providing peer-reviewed precedent for tier-based reasoning in computational pharmacology [70,96]. The author’s interpretation is that these principles offer conceptual, rather than prescriptive, support for the risk-tiered approach to AI validation proposed in Section 4. This extension does not represent an agency position.

### 5.3. Lifecycle Governance and Model Drift

**Figure 5:**
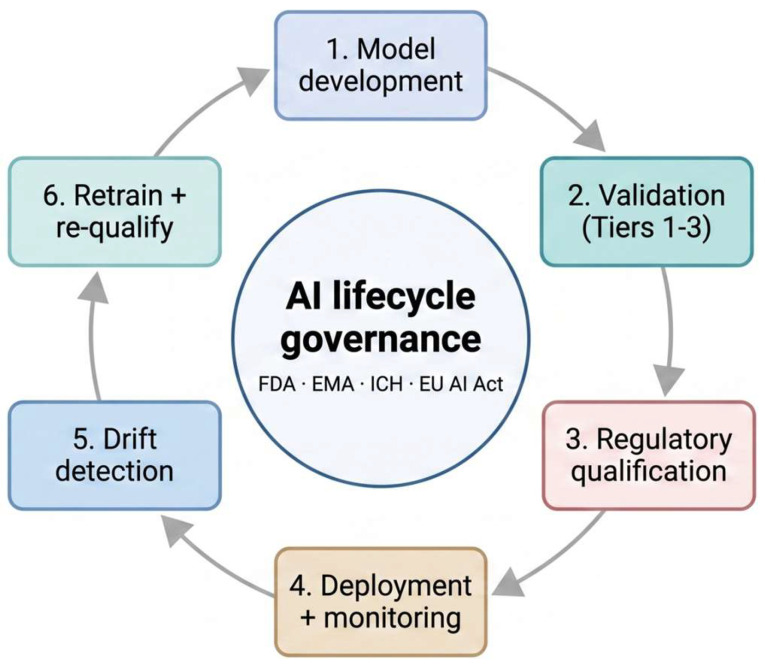
AI lifecycle governance as a closed-loop system. Each transition between stages functions as a checkpoint, whereby predefined acceptance criteria must be satisfied prior to the system’s progression. Detection of drift at stage 5 initiates retraining at stage 6 and subsequent requalification, in accordance with the associated risk tier of the intended application. This framework aligns with established Good Manufacturing Practice (GMP) lifecycle management for analytical methods, while also addressing specific failure modes of machine learning systems, namely dataset shift, concept drift, and upstream dependency change.

Compared to static analytical instruments, machine learning systems experience continuous development. Variations in performance may result from the accumulation of training data, shifts in operational distribution, or autonomous updates to upstream dependencies such as reference databases and preprocessing pipelines, which are external to the model itself. Therefore, effective lifecycle management requires the implementation of predefined monitoring thresholds, strict version control, and retraining policies in accordance with Good Manufacturing Practice (GMP) standards for analytical methods [17,78]. Figure 5 illustrates the lifecycle as a closed-loop process, where each transition represents a potential regulatory checkpoint.

Global development initiatives require coordination across jurisdictions; disparate evidentiary standards can lead to fragmentation and redundant validation efforts. Harmonization through ICH working groups would promote the adoption of standardized templates for algorithmic transparency, uncertainty reporting, and benchmarking criteria, thereby minimizing the risk of inconsistent evaluation of equivalent evidence packages by different agencies [17,18,97].

### 5.4. Transparency, Explainability, and Documentation

Post hoc interpretability methods offer limited insights into model behavior and do not serve as substitutes for empirical validation [98]. Structured reporting mechanisms, such as model cards, data sheets, and context-of-use specifications, formalize the documentation of intended applications, performance metrics, and limitations [98,99]. For AI systems whose outputs influence regulatory decisions, the documentation process is not merely administrative overhead; it is a primary deliverable, as it determines whether evidence generated today can be reliably interpreted and audited years hence, despite potential changes in the model, training data, and computational environment.

### 5.5. Relationship to Existing Reporting and Governance Frameworks

The four-tier validation ladder proposed in Section 4 is designed to operate in conjunction with, rather than as a substitute for, several established machine learning reporting and governance frameworks. The FDA Good Machine Learning Practice (GMLP) guiding principles, collaboratively issued with Health Canada and the United Kingdom MHRA, delineate 10 overarching expectations for AI/ML-enabled medical software, including the use of representative training datasets, performance assessment across relevant subgroups, transparency to end users, and post-deployment surveillance. The U.S. National Institute of Standards and Technology AI Risk Management Framework (NIST AI RMF) delineates a higher-level governance vocabulary focused on the functions of GOVERN, MAP, MEASURE, and MANAGE. Reporting standards targeting clinical prediction modeling, such as TRIPOD-AI and the recently revised TRIPOD+AI statement [100], specify the disclosures required when reporting prediction models that use regression or machine learning techniques. Furthermore, CONSORT-AI and SPIRIT-AI extend the standards for trial reporting to interventions that incorporate AI components. More closely associated with the molecular sciences discussed in this article, the DOME recommendations outline baseline reporting expectations for supervised machine learning applications in the life sciences. The MI-CLAIM checklist addresses clinical AI, and the MAQC/SEQC consortium has established practical standards to ensure the reproducibility of high-throughput biological data analysis.

These frameworks differ from the present proposal in scope and the questions they address. GMLP, NIST AI RMF, and the EU AI Act are governance-focused, primarily addressing the query, “what kinds of controls and oversight should an AI system be subjected to?” Conversely, TRIPOD+AI, CONSORT-AI, SPIRIT-AI, DOME, MI-CLAIM, and MAQC/SEQC are oriented towards reporting and primarily answer, “what should be disclosed when a study or model is published?” The four-tier validation ladder proposed herein is directed towards evidentiary considerations and addresses a distinct question: “How much computational, experimental, and translational evidence is necessary before a specific AI prediction can be appropriately employed for a particular decision?” Consequently, these tiers complement rather than duplicate existing frameworks. An AI/ML application may be reported in accordance with TRIPOD+AI [100] or DOME, governed under GMLP and NIST AI RMF, regulated as a high-risk AI system under the EU AI Act, and simultaneously classified as Tier 2 or Tier 3 evidence within this framework when utilized to support specific decisions in drug discovery. The ladder serves as a common language for the evidentiary level of an AI prediction, operating alongside procedural and documentation standards established by these other frameworks. In instances where the ladder intersects with existing standards, for example, in advocating calibrated uncertainty, drift monitoring, and lifecycle governance, the existing standards should be regarded as authoritative, with the ladder providing a means to map their compliance to the level of evidentiary support an AI prediction offers for translational decision-making.

### 5.6. Ethical, Equity, and Sustainability Considerations

Bias inherent in datasets concerning chemical or patient populations propagates through artificial intelligence (AI) pipelines, potentially leading to predictions that vary across different demographic or chemotype subgroups. Mitigation measures encompass diversification of datasets, the implementation of fairness-aware learning objectives, and conducting independent bias audits. Computational costs present a separate issue: large-scale molecular dynamics simulations, diffusion training, and foundation-model development demand substantial energy consumption. It is increasingly regarded as best practice within the spheres of responsible AI and environmental accountability to report computational energy consumption and estimated CO_2_-equivalent emissions alongside performance metrics [101], and this should be routinely incorporated into validation documentation.

## 6. Clinical Case Studies Through the Validation Lens

The subsequent clinical-stage examples demonstrate the correlation between the validation hierarchy and actual programs. Each case has been carefully selected to underscore a particular level of evidence and is presented solely for illustrative purposes; the causal attribution of clinical success or failure to any specific modeling decision cannot generally be ascertained based on publicly available information and is not asserted herein.

### 6.1. Tier 2–3 Success: Insilico Medicine INS018_055 (TNIK, IPF)

Insilico Medicine’s anti-fibrotic TNIK inhibitor INS018_055 (rentosertib) has advanced to a Phase IIa clinical trial for idiopathic pulmonary fibrosis (NCT05938920) [102]. A dual-artificial intelligence methodology identified TNIK as a target through transcriptomic analysis, while a generative system was utilized to design optimized molecules. This approach demonstrates that Tier 2 (benchmark-level) structural prediction, combined with Tier 3 (prospective experimental) medicinal chemistry validation, can underpin successful Investigational New Drug (IND)-enabling campaigns when the target maintains a druggable dominant conformation. The methodology does not depend on ensemble predictions, and its progression from preclinical to clinical stages serves to validate the underlying approach rather than the ensemble methods themselves; rather, it affirms the efficacy of artificial intelligence-augmented target identification and lead optimization for a well-characterized target.

### 6.2. Tier 2 Proof-of-Concept: Insilico CDK20 for Hepatocellular Carcinoma

In a separate initiative, Insilico Medicine utilized AlphaFold-predicted structures of cyclin-dependent kinase 20 (CDK20), a target lacking experimental structural data, to identify a sub-micromolar hit for hepatocellular carcinoma from an initial set of seven synthesized compounds. This was followed by a series of iterative optimizations documented over an extended period [103]. This demonstrates that AI-generated structures can form a basis for effective medicinal chemistry efforts targeting proteins without experimental structural information, provided that iterative generative refinement is employed. The program primarily relied on Tier 2 evidence for structural prediction and Tier 3 evidence for medicinal chemistry.

### 6.3. A Systematic Caveat: AlphaFold2 Kinase Conformational Bias

A comprehensive analysis of kinase structures predicted by artificial intelligence revealed that AlphaFold2 prefers active-state (DFG-in) conformations, thereby reflecting the overrepresentation of these conformations in experimental databases [104]. Given that numerous clinically effective kinase inhibitors target inactive (DFG-out or αC-out) conformations, this bias constitutes a significant limitation for structure-based drug design, particularly concerning Type-II and allosteric kinase therapeutics. To address this bias, reducing the depth of multiple sequence alignments during AlphaFold2 predictions has proven effective, while ensemble-emulation techniques (Section 2.3) provide additional mitigation strategies. This underscores the importance of understanding that Tier 2 benchmark performance in CASP-style structural accuracy does not necessarily equate to suitability for practical applications, as the scoring metric and the clinically relevant metric are not invariably aligned.

### 6.4. Tier 2–3 Hypothesis Not Yet at Tier 4: BenevolentAI Computational Hypothesis for Baricitinib in ALS

BenevolentAI employed its knowledge-graph platform to investigate amyotrophic lateral sclerosis (ALS) and generated, as a computational hypothesis, the approved JAK inhibitor baricitinib as a candidate for drug repurposing, based on predicted involvement of JAK/STAT signaling in neuroinflammatory and motor-neuron pathologies. It was posited that the drug’s capacity to penetrate the central nervous system and its anti-inflammatory properties provided a rationale for translational application [105]. This instance is cited here as an example of hypothesis generation rather than as validated translational success, and the two distinct applications of the BenevolentAI knowledge graph platform should not be conflated. The extensively cited COVID-19 repurposing hypothesis for baricitinib [106] subsequently progressed through randomized controlled trials and is recognized as a Tier 4–validated outcome within the framework of this article: a computationally generated hypothesis that has been substantiated by clinical efficacy data and integrated into clinical practice. Conversely, the ALS application has not achieved Tier 4 status. As of early 2026, it remains a published computational and preclinical hypothesis; no Phase 2/3 randomized efficacy trial of baricitinib in ALS has been documented in the peer-reviewed literature. Therefore, the ALS case is not characterized in this article as a clinical failure, since no sufficiently powered efficacy trial has been conducted, but rather as an example of a Tier 2 to Tier 3 computational and preclinical rationale that has yet to generate the clinical evidence necessary for Tier 4 qualification under the proposed framework. This illustrative case underscores that Tier 2 and Tier 3 computational rationales for repurposing hypotheses do not substitute for Tier 4 clinical and translational validation; they identify candidates worthy of clinical evaluation but do not establish efficacy. A genuine clinical failure outcome in the same therapeutic area is exemplified by the CNM-Au8 program, which was evaluated in the Phase 2 RESCUE-ALS trial [107] and failed to achieve its primary neurophysiological endpoint, broadly demonstrating that mechanistically plausible candidates in ALS frequently do not succeed in randomized clinical trials, regardless of computational identification. The two cases exemplify different positions on the validation continuum: the baricitinib-in-ALS hypothesis as Tier 2–3 evidence not yet tested at Tier 4, and CNM-Au8 in RESCUE-ALS as Tier 4 evidence with a negative outcome. The attribution of causality for the CNM-Au8 outcome based on specific modeling choices cannot be confirmed from publicly available information and is not claimed herein. Plausible contributors to translational challenges in ALS include patient heterogeneity, variability in blood–brain-barrier penetration, disease-stage-dependent target engagement, and discrepancies between predicted and actual target occupancy in the affected tissue.

### 6.5. Oncology as a Current Proving Ground

Oncology represents the most active therapeutic domain within AI-enabled drug discovery. A comprehensive landscape analysis of AI-native biotechnology firms reported by Jayatunga et al. [108] indicated that their clinical pipelines are disproportionately concentrated in oncology, and that AI-discovered drugs in that survey had demonstrated Phase I success rates surpassing historical industry averages in preliminary assessments. That analysis carries important limitations that should be made explicit. The cohort it analyzes is small in absolute terms; the period covered captures only early Phase I outcomes and Phase II data are still maturing; the “AI-discovered” label is applied heterogeneously across companies and ranges from candidates whose lead identification used AI to candidates whose entire discovery cycle was AI-driven; and a substantial fraction of the underlying outcome data is sourced from company disclosures rather than independent registries, introducing reporting bias. Independent reviews of AI-driven oncology discovery, including the structure-prediction-focused analysis by Qiu et al. [109], converge on a similar conclusion: the technology is enabling more rapid early-stage progression, but translational outcomes remain to be confirmed in adequately powered later-phase trials. The encouraging Phase I observations should therefore be interpreted as preliminary and as a hypothesis-generating signal rather than as established evidence that AI-discovered oncology candidates outperform conventionally discovered ones. The focus on oncology is supported by favorable enabling conditions: target heterogeneity fuels demand for novel modalities, resistance-driven conformational plasticity is well understood, kinase signaling networks are central and extensively researched, and multi-omics datasets establish robust links between molecular features and clinical outcomes [110]. AI-powered integration of multi-omics data employs graph-based learning to identify candidate targets and patient stratification across genomic, transcriptomic, and proteomic layers [111]. AI-derived synergistic drug combinations have been experimentally prioritized at rates aligned with predictive models, although their clinical validation remains ongoing [112]. Disclosures from Isomorphic Labs, Recursion, Exscientia, and Schrödinger suggest that oncology candidates are likely among the first AI-designed drugs to advance to pivotal trials across multiple platforms [108,113]. To date, none of these programs has resulted in an approved AI-designed drug. The framework discussed in this article applies the same Tier 4 evidentiary standards to oncology approvals as to any other therapeutic area.

## 7. Conclusions and Recommendations

The assertion that artificial intelligence (AI) in drug discovery is limited by its inability to model protein ensembles was accurate for several years and has begun to be addressed over the past 18 months. BioEmu, AlphaFlow, DiG, Boltz-2, Chai-1, SuperWater, MISATO, and their counterparts have begun to address the fundamental gaps previously identified, although some of these claims are based on preprints that have yet to undergo peer review. Collectively, the existing evidence indicates that the translational bottleneck is shifting from AI capabilities to the qualification of AI evidence for decision-making with therapeutic implications. Nevertheless, this transition remains incomplete and highly context-dependent.

This article has argued for the following:

- The evidentiary burden ought to be proportionate to the degree of risk implicated. A hierarchical framework with four tiers, comprising internal reproducibility, benchmark robustness, prospective experimental validation, and clinical and translational calibration, functions as a structured scaffold for aligning evidence with potential outcomes. No single tier is adequate independently; instead, convergence across these levels underpins the validity of substantial translational claims.
- Ensemble-aware artificial intelligence constitutes a methodological classification rather than a universal prerequisite. For targets that are conformationally rigid, well-characterized, and supported by comprehensive structure–activity relationship (SAR) data, static structure prediction employing machine learning-augmented scoring may be deemed adequate. Ensemble methodologies are justified in circumstances where target conformational flexibility is mechanistically pertinent, where binding kinetics are vital, or where the therapeutic hypothesis relies on a minor-population state.
- Uncertainty quantification is an integral component rather than a supplementary appendix. Every AI-generated prediction that influences subsequent decision-making processes must incorporate calibrated uncertainty. Additionally, this uncertainty must be propagated through PBPK, QSP, and trial-simulation frameworks when predictions are applied to Tier 4 use cases.
- Lifecycle governance constitutes the primary operational challenge. Machine learning systems undergo continuous evolution; evidence packages deemed valid at deployment may become obsolete as data, dependencies, and operational environments change. The implementation of drift detection, predefined retraining triggers, and version control are essential deliverables, rather than solely administrative burdens.
- Harmonizing regulatory frameworks would facilitate the responsible deployment of technologies. The existence of divergent evidentiary standards among agencies such as the FDA, EMA, PMDA, NMPA, and TGA poses risks of duplication and fragmentation. Achieving ICH-level harmonization of criteria related to algorithmic transparency, uncertainty reporting, and benchmarking would mitigate these issues without compromising standards.
- The publication of failures holds the same importance as that of successes. The imbalance between reported achievements in AI-driven drug discovery and the unreported failures leads to an inflated perception of reliability and misguides resource distribution. Academic journals, collaborative consortia, and regulatory bodies ought to implement systematic mechanisms for the reporting of negative prospective outcomes.

The fundamental transition involves advancing from deterministic structural inference to a probabilistic, multi-scale modeling methodology incorporated within regulatory frameworks that explicitly consider uncertainty. Artificial Intelligence serves as an integrative tool within a broader scientific domain, rather than as an oracle. AI-supported drug design will evolve through probabilistic, mechanistically grounded translational systems that link molecular predictions with clinical reliability, supported by evidence consistent with the significance of the outcomes.

## Figures and Tables

**Table 1:** Contemporary AI model categories relevant to drug discovery (2024–2026).

Category	Representative Models	Output	Key Limitation
Static structure prediction	AlphaFold2/3, RoseTTAFold, ESMFold	Dominant conformation + pLDDT	Single state; weak on cryptic pockets
Complex prediction	AlphaFold3, Boltz-1, Chai-1	Multi-chain structure	Pose reliability degrades out-of-distribution
Ensemble emulation	BioEmu, AlphaFlow, DiG	Approximate equilibrium distribution	No native ligand/membrane; domain-limited
Joint structure + affinity	Boltz-2, NeuralPLexer	Structure + ΔG_bind_ with uncertainty	Variable performance across protein classes
Explicit-solvent prediction	SuperWater, HydraProt, GalaxyWater-CNN	Water positions with confidence	Trained on crystal waters; dynamic hydration approximate
Machine-learned potentials	MACE, Allegro, ANI, SchNet	QM-accuracy forces/energies	Transferability outside training in chemistry
Generative molecular design	REINVENT, MolMIM, Chroma, RFdiffusion	De novo molecules or proteins	Synthesizability, physicality, surrogate misalignment
MD-augmented datasets	MISATO, ATLAS, PLINDER	Training/benchmark resources	Coverage biases in source PDB

**Table 2:** Representative AI use cases are mapped to the minimum validation tier and persistent failure mode.

AI Use Case	Risk Level	Minimum Tier	Persistent Failure Modes
Structure prediction for exploratory analysis	Low	Tier 2	Low confidence in loops/IDRs single-state output cofactor effects absent
Ensemble emulation for hypothesis generation	Low–Medium	Tier 2	Domain-limited transferability no ligand/membrane distributional shift
AI docking for virtual screening	Medium	Tier 2–3	Pose chemical validity novel-chemotype failure implicit-solvent bias
Generative molecular design (lead discovery)	Medium–High	Tier 3	Mode collapse surrogate misalignment synthesizability physicality
RBFE for lead optimization	Medium	Tier 3	Force-field inaccuracy sampling scaffold hops unreliable
AI-driven antibody/protein design	High	Tier 4	Aggregation CDR dynamics glycosylation immunogenicity
AI-derived PK parameters → PBPK	High	Tier 4	Uncertainty propagation interindividual variability model drift
AI-informed clinical dose selection	High	Tier 4	Context-of-use drift population coverage regulatory acceptance

Glossary of failure modes referenced in Table 2. Mode collapse: a generative model produces a narrow, repetitive subset of plausible outputs and fails to cover the full diversity of valid candidates. Surrogate misalignment: the optimization objective used to score generated molecules (a learned surrogate or proxy reward) does not faithfully reflect the true biological or pharmacological objective, so models maximize a proxy that does not translate to the desired outcome. Synthesizability: the predicted molecule cannot be made by accessible synthetic chemistry, or can be made only with an effort disproportionate to the project. Physicality (or physical plausibility): the predicted three-dimensional structure or pose violates basic physical and chemical constraints such as bond lengths, valence, ring planarity, or steric overlap, regardless of nominal scoring accuracy. Distributional shift: the test inputs come from a different statistical distribution than the training inputs, leading to degraded accuracy and calibrated uncertainty estimates, often undetected by in-distribution validation. Context-of-use drift: the operational context in which a model is applied (target class, patient population, assay platform, regulatory question) gradually departs from the context for which the model was qualified, eroding the validity of its predictions over time. Model drift: changes in upstream data sources, software dependencies, or retraining cycles produce silent shifts in model behavior between releases, requiring active monitoring and re-qualification.

## Data Availability

No new data were created in this study. Data sharing is not applicable to this article.
